# 

**Published:** 1907-01

**Authors:** 


Sixty-second Year.

Vol. LXII. JANUARY, 1907. No. 6

### BUFFALO MEDICAL JOURNAL

Established 1845 by AUSTIN FLINT, M. D.

LIBRARY
JAN 1907

EDITOR:
WILLIAM WARREN POTTER, M. D.

ASSISTANT EDITORS:
WILLIAM C. KRAUSS, M. D. NELSON W. WILSON, M. D.

ASSOCIATE EDITORS:
JAMES WRIGHT PUTNAM, M. D. ERNEST WENDE, M. D.
JOHN PARMENTER, M. D. JOHN A. MILLER, Ph. D.
HARVEY R. GAYLORD, M. D MAUD J. FRYE, M. D.
JOHN A. RAFTER, M. D.

